# Isolation of T cell receptors specifically reactive with mutated tumor associated antigens

**DOI:** 10.1186/2051-1426-2-S3-P33

**Published:** 2014-11-06

**Authors:** Maria Parkhurst, Paul Robbins, Steven Rosenberg

**Affiliations:** 1 NIH/NCI/Surgery Branch, Bethesda, MD, United States

## Background and hypotheses

The adoptive transfer of tumor infiltrating lymphocytes (TIL) can mediate the regression of metastatic melanoma [1]. In addition, the adoptive transfer of lymphocytes expressing T cell receptors (TCRs) specifically reactive with antigens expressed on melanoma cells can mediate tumor regression [2]. Many T cells from TIL recognize mutated antigens expressed only on the autologous patient's tumor cells [3]. Therefore, we have attempted to isolate TCRs reactive with unique mutated antigens so that we may eventually treat patients with autologous T cells that have been genetically modified to express those TCRs.

## Study design and methods

Exome sequencing and RNA sequencing were used to identify mutated antigens that are highly expressed in tumors. In this study, we used 2 methods to isolate TCRs reactive with mutated antigens as follows:

1. TIL were stimulated overnight with autologous dendritic cells (DCs) electroporated with in vitro transcribed RNAs encoding mutations. CD3+ CD8+ T cells that had upregulated CD137 after stimulation were then FACS sorted and expanded in vitro with anti-CD3 and IL2.

2. Peptides encompassing mutations with high predicted binding affinities to HLA-A*0201 were used to stimulate peripheral blood lymphocytes (PBL) from autologous patients expressing this HLA molecule. PBL were stimulated in vitro with peptide-pulsed autologous mature DCs and restimulated 7-10 days later with peptide-pulsed autologous PBMCs. T cells that upregulated CD137 after stimulation with 239 or COS cells expressing HLA-A*0201 and the mutation were then sorted and expanded in vitro with anti-CD3 and IL2. Recognition of appropriate target cells by the resulting T cell populations was evaluated on the basis of IFNγ secretion and CD137 expression. For populations which appeared to be enriched for T cells capable of recognizing mutated antigens, TCR α and β chain sequences were identified using 5' RACE, and retroviruses encoding those TCRs were used to transduce PBL.

## Results and conclusions

Using these techniques, we identified, enriched, and expanded T cell populations that recognized mutated tumor associated antigens. We also identified dominant TCR α and β chains in these enriched populations. By using retroviruses encoding the dominant TCRs to transduce human PBL, we demonstrated that these TCRs mediated recognition of the expected tumor associated mutated antigens (Table 1). We are currently attempting to develop clinical reagents to treat patients with TCRs that recognize unique mutations on their autologous tumor cells.

**Table 1:** IFNγ secretion (pg/ml) by TCR transduced PBL

TCR source^a,b^	TCR α/β^c^	media	T2+ HBV^d ^peptide	T2+ mutated AHNAK^e ^peptide	T2+ wild type AHNAK peptide	T2+ mutated SRPX^f ^peptide	T2+ wild type SRPX peptide	Allogeneic melanoma (A2+)	Autologous melanoma (A2+)
peptide stimulated PBL^a^	TRAV12-2*01/ TRBV2*01	118	167	**>10000**	698	220	244	85	**9784**
peptide stimulated PBL	TRAV19*01/ TRBV12-4*01	90	100	150	154	**>10000**	246	60	**9715**
peptide stimulated PBL	TRAV3*01/ TRBV2*01	84	84	121	105	**>10000**	217	34	**>10000**
CD137 FACS sorted TIL^b^	TRAV29/DV5*01/ TRBV5-6*01	126	134	139	189	**>10000**	183	55	**>10000**

^a ^CD8+ PBL from patient 3713 were stimulated in vitro with mature autologous DCs pulsed with mutated peptides predicted to bind with high affinities to HLA-A*0201 and were restimulated twice with autologous peptide-pulsed PBMCs. Recognition of relevant target cells was evaluated on the basis of IFNγ secretion after overnight coculture. 2 peptides, one derived from a mutation in the AHNAK protein and one derived from a mutation in the SRPX protein, stimulated T cells that specifically recognized peptide, COS7 cells expressing HLA-A*0201 that had been transfected with the relevant minigene, and the autologous tumor cell line.

^b ^TIL from patient 3713 were cocultured overnight with autologous DCs electroporated with in vitro transcribed (IVT) RNA encoding a fragment of the mutated SRPX protein. CD3+ CD8+ 41BB+ cells were sorted by FACS and expanded in the presence of allogeneic feeder cells, α-CD3, and IL2. The resulting T cell population specifically recognized peptide, COS7 cells expressing HLA-A*0201 that had been transfected with the relevant minigene, and the autologous tumor cell line.

^c ^TCR α and β chains in T cell populations were identified by 5' RACE using degenerate constant region primers and were cloned into retroviral vectors. These were then used to transduce PBL from patient 3713, and the function of the resulting genetically modified T cells was evaluated on the basis of IFNγ secretion after overnight coculture.

^d ^HBV: hepatitis B core virus peptide used as a negative control with high binding affinity to HLA-A*0201.

^e ^AHNAK: neuroblast differentiation-associated protein, also known as desmoyokin.

^f ^SRPX: sushi repeat-containing protein.

## Consent

Written informed consent was obtained from the patient for publication of this abstract and any accompanying images. A copy of the written consent is available for review by the Editor of this journal.
